# TNF‐α/IFN‐γ synergy amplifies senescence‐associated inflammation and SARS‐CoV‐2 receptor expression via hyper‐activated JAK/STAT1

**DOI:** 10.1111/acel.13646

**Published:** 2022-05-30

**Authors:** Renuka Kandhaya‐Pillai, Xiaomeng Yang, Tamar Tchkonia, George M. Martin, James L. Kirkland, Junko Oshima

**Affiliations:** ^1^ Department of Laboratory Medicine & Pathology University of Washington Seattle Washington USA; ^2^ Robert and Arlene Kogod Center on Aging Mayo Clinic Rochester Minnesota USA; ^3^ Department of Physiology Mayo Clinic Rochester Minnesota USA; ^4^ Department of Medicine Mayo Clinic Rochester Minnesota USA

**Keywords:** ACE2, COVID‐19, cytokines, DPP4, inflammation, JAK–STAT, SARS‐COV‐2 receptor, senescence

## Abstract

Older age and underlying conditions such as diabetes/obesity or immunosuppression are leading host risk factors for developing severe complications from COVID‐19 infection. The pathogenesis of COVID‐19‐related cytokine storm, tissue damage, and fibrosis may be interconnected with fundamental aging processes, including dysregulated immune responses and cellular senescence. Here, we examined effects of key cytokines linked to cellular senescence on expression of SARS‐CoV‐2 viral entry receptors. We found exposure of human umbilical vein endothelial cells (HUVECs) to the inflammatory cytokines, TNF‐α + IFN‐γ or a cocktail of TNF‐α + IFN‐γ + IL‐6, increased expression of ACE2/DPP4, accentuated the pro‐inflammatory senescence‐associated secretory phenotype (SASP), and decreased cellular proliferative capacity, consistent with progression towards a cellular senescence‐like state. IL‐6 by itself failed to induce substantial effects on viral entry receptors or SASP‐related genes, while synergy between TNF‐α and IFN‐γ initiated a positive feedback loop via hyper‐activation of the JAK/STAT1 pathway, causing SASP amplification. Breaking the interactive loop between senescence and cytokine secretion with JAK inhibitor ruxolitinib or antiviral drug remdesivir prevented hyper‐inflammation, normalized SARS‐CoV‐2 entry receptor expression, and restored HUVECs proliferative capacity. This loop appears to underlie cytokine‐mediated viral entry receptor activation and links with senescence and hyper‐inflammation.

## Graphical abstract

TNF‐α and IFN‐γ synergistically enhanced expression of SARS‐CoV‐2 viral entry receptors, amplified senescence‐associated inflammation, and initiated a positive feedback loop via hyper‐activation of the JAK/STAT1 pathway in endothelial cells. Inhibition of JAK/STAT with ruxolitinib or antiviral remdesivir normalised ACE2/DDP4 expression and controlled hyper‐inflammation. Therapies with JAK inhibitors or drugs targeting JAK/STAT signaling could alleviate complications of cytokine storm in severe COVID‐19 infection.

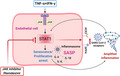

## 1 INTRODUTION

COVID‐19 infection, caused by Severe Acute Respiratory Coronavirus‐2 (SARS‐CoV‐2), has more serious consequences for the elderly and chronically‐ill than previously healthy younger individuals (Clark et al., 2020; Davies et al., 2020). The severity of COVID‐19 illness is increased, even in younger individuals, with such conditions as diabetes, obesity, hypertension, immunosuppression, cancers, and chronic respiratory diseases (Verdoorn et al., 2021; Wissler Gerdes et al., 2022). Hyper‐inflammation or cytokine storm is considered a key pathological threat to patients with COVID‐19 and often portends poor clinical outcomes (Mehta et al., 2020; Tisoncik et al., 2012). Excessive production of inflammatory factors including IL‐6, TNF‐α, IL‐1, GM‐CSF, IP‐10, and MCP‐1 are important predictors of disease progression, severity, and mortality in COVID‐19 infection (Del Valle et al., 2020; Huang et al., 2020; Moore & June, 2020). Cytokine storm closely resembles the inflammatory phenotype that can be linked to cellular senescence and that contributes to the pathogenesis of multiple diseases (Coppé et al., 2010; Ye et al., 2020). The underlying mechanisms through which the elderly and those with chronic conditions are adversely affected by COVID‐19 infection and how presence of senescent cells and their associated pro‐inflammatory SASP influence severity of COVID‐19 need to be elucidated.

Inflammation is an important protective innate immune response that is triggered within hosts by infection or injury. Cytokines secreted from immune cells regulate inflammatory processes in which the onset, development, and resolution of inflammation are tightly controlled by a sequence of molecular events (Chen et al., 2017). Successful and effective resolution of inflammation is essential for preventing progression of acute inflammation into a persistent, detrimental, chronic inflammatory state. During aging, systemic, chronic, low grade, sterile inflammation, or “inflamm‐aging”, coupled with declines in immune function, or “immuno‐senescence”, contributes to accumulation of senescent cells and development of age‐related pathologies (Franceschi et al., 2000). Analogously, chronic inflammation is an integral part of aging and cellular senescence and is a potential trigger for a number of chronic diseases; senescent cells themselves can act as a source of chronic inflammation through their SASP (Coppé et al., 2010; Freund et al., 2010; Pignolo et al., 2020; Song et al., 2020; Tchkonia et al., 2021; Tchkonia & Kirkland, 2018; Wissler Gerdes et al., 2020). Many SASP factors, including IL‐6, TNF‐α, IL‐1, G‐CSF, IP‐10, MCP‐1, and IFN‐γ are also major components of SARS‐CoV‐2‐related cytokine storm. The inflammatory cytokines, TNF‐α, IFN‐γ, and IL‐6, function as mediators of inflammation and are known to accelerate and reinforce cellular senescence via positive feedback loops (Acosta et al., 2008; Freund et al., 2010; Kandhaya‐Pillai et al., 2017). Furthermore, SARS‐CoV‐2 can cause non‐senescent cells to become senescent (Camell et al., 2021; Lee et al., 2021; Tripathi et al., 2021). The hallmarks of aging, inflammation, and multiple chronic diseases are highly interrelated (Tchkonia et al., 2021; Wissler Gerdes et al., 2021) and directly correlate with pathological outcomes of severe COVID‐19. It may be the case that underlying chronic inflammation and senescent cell burden in the elderly or chronically ill can fuel cytokine storm and so cause severe, serious consequences of SARS‐CoV‐2 infection (Camell et al., 2021; Tripathi et al., 2021; Wissler Gerdes et al., 2021).

The receptor binding domain of SARS‐CoV‐2 spike protein (S) interacts with cell surface receptors for viral entry to infect human cells. The S protein of SARS‐CoV‐2 attaches to angiotensin‐converting enzyme‐2 (ACE2) for host cell entry (Hoffmann et al., 2020). ACE2 has been recently identified as an interferon‐stimulated gene and is widely distributed across multiple cell types, including human epithelial, adipose, lung, liver, heart, and kidney cells, enterocytes in the small intestine, and endothelial cells (Ziegler et al., 2020). In addition to ACE2, SARS‐CoV‐2 can utilize other co‐receptors and binding factors for entering host target cells. Dipeptidyl‐peptidase IV (DDP4), also referred as to CD26, is a binding receptor for Middle East Respiratory Syndrome coronavirus (MERS‐CoV) that enables viral entry (Raj et al., 2013). DPP4 is expressed on the cell surface of senescent cells and is known to regulate inflammation (Kim et al., 2017; Trzaskalski et al., 2020). Recently, DPP4 has been identified as a binding target of SARS‐CoV‐2 S protein and may be co‐expressed with ACE2 (Li et al., 2020; Vankadari & Wilce, 2020). Deregulated expression of both ACE2 and DPP4 are associated with old age, inflammation, diabetes, obesity, hypertension, and other common age‐associated conditions, all of which are reported to increase risk for severe complications from COVID‐19 infection (Valencia et al., 2020). It is conceivable that enhanced expression of these receptors contributes to enhanced viral entry and disease severity in the older population and those with comorbid conditions.

There is pathological evidence that SARS‐CoV‐2 infection can be linked to endothelial cell dysfunction, fibrosis, and thrombosis (Bonaventura et al., 2021; Huertas et al., 2020). Endothelial cell injury coupled with excessive inflammation appears to be a key driver of COVID‐19 severity (Jin et al., 2020). Because TNF‐α, IFN‐γ, and IL‐6 are considered to be markers of both cellular senescence and COVID‐19 cytokine storm, understanding the roles and mechanisms of effects of these cytokines on COVID‐19 receptor expression is important in order to identify specific therapeutic targets to prevent or alleviate complications of this disease. Recently, we reported that senolytic drugs reduce inflammation, senescent cell burden, and complications from infection of older mice by a β‐coronavirus, mouse hepatitis virus, which is related to SARS‐CoV‐2 (Camell et al., 2021). Here, we show that senescent HUVECs have increased expression of the SARS‐CoV‐2 uptake receptors, ACE2 and DPP4, compared to non‐senescent cells. Our data reveal that the inflammatory cytokines, TNF‐α and IFN‐γ, synergistically enhance activation of ACE2 and DPP4 receptors in endothelial cells via the JAK/STAT pathway.

## 2 RESULTS

### 2.1 Evidence for SARS‐CoV‐2 receptor expression in senescent endothelial cells

**FIGURE 1:**
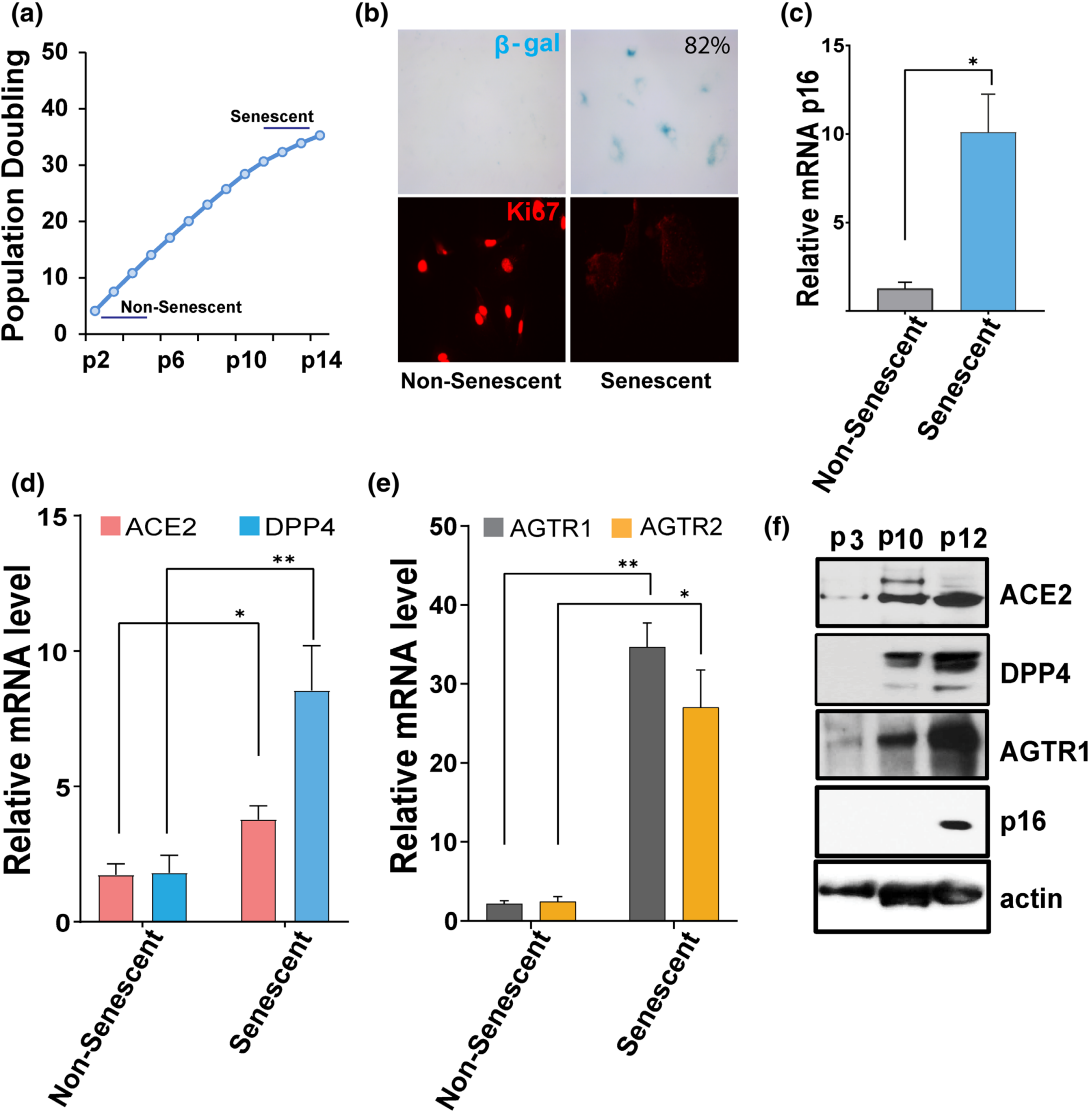
SARS‐CoV‐2 viral entry receptor expression is increased in senescent HUVECs. (a) Population doubling times of HUVECs. “Young” (passage 2–5) HUVECs were serially cultured until they entered proliferative arrest at passage 11–14 (Senescent). (b) Non‐senescent (passage 2) and senescent (passage 14) cells were stained for SA‐β‐gal activity (blue cells) and the proliferative marker Ki67 (in red). (c) Real‐time PCR analysis for the senescence marker *p16* ^*INK4a*^ in non‐senescent and senescent cultures. (d, e) quantification of ACE2, DPP4, AGTR1, and AGTR2 mRNA in non‐senescent and senescent cells. GAPDH was used as an internal control. Error bars show mean ± SD, *p < 0.05, **p < 0.01, ***p < 0.001, Student's T‐test. f) Western blots for ACE2, DPP4, AGTR1, p16, and actin in non‐senescent (passage 3) and senescent cells (passage 10–12). All real‐time PCR reactions were conducted in triplicate. Data are representative of three (a–d) or two (e) independent experiments

The initiation of viral infection depends on binding to host cell surface receptors for viral entry (Maginnis, 2018). We assessed whether viral entry receptors are present on senescent HUVECs and whether expression differs from non‐senescent cells. Senescence of HUVECs was induced by serially passaging non‐senescent (passage 1–3) HUVECs until their proliferative capacity had decreased by passage 11–14 (Figure 1a). Senescent HUVECs displayed flattened and enlarged morphology, increased senescence‐associated β‐galactosidase (SA‐β‐gal), reduced expression of the proliferative marker Ki67, and increased expression of *p16* ^*INK4a*^ and SASP factors (Figure 1b, c, S1). We next examined the expression patterns of the SARS‐CoV‐2 receptors, ACE2 and DPP4, in non‐senescent and senescent HUVECs. Compared to non‐senescent HUVECs, ACE2 expression in senescent cells was increased by threefold, while DPP4 was even more abundant: more than tenfold higher in senescent than non‐senescent cells (Figure 1d). Since senescent cell burden increases with aging, these data suggest that the lower incidence of severe infection in children compared to aged adults may in part be related to lower expression of these viral entry receptors. ACE2 regulates angiotensin II (ANGII) signaling and ANGII receptors types 1 and 2 (AGTR1 and AGTR2, respectively), each of which can play a role in controlling inflammation. We noted that expression of both AGTR1 and AGTR2 was higher in senescent than non‐senescent cells (Figure 1e). Moreover, high levels of ACE2, DPP4, and AGTR1 proteins in senescent cells correlated with increased *p16* ^*INK4a*^ expression (Figure 1f). Increased expression of SARS‐CoV‐2 receptors in senescent endothelial cells may contribute to the increased risk of severe infection in the elderly and those with disorders associated with increased senescent cell burden.

### 2.2 TNF‐α/IFN‐γ synergism enhances SARS‐CoV‐2 receptor activation and induces proliferative arrest

**FIGURE 2:**
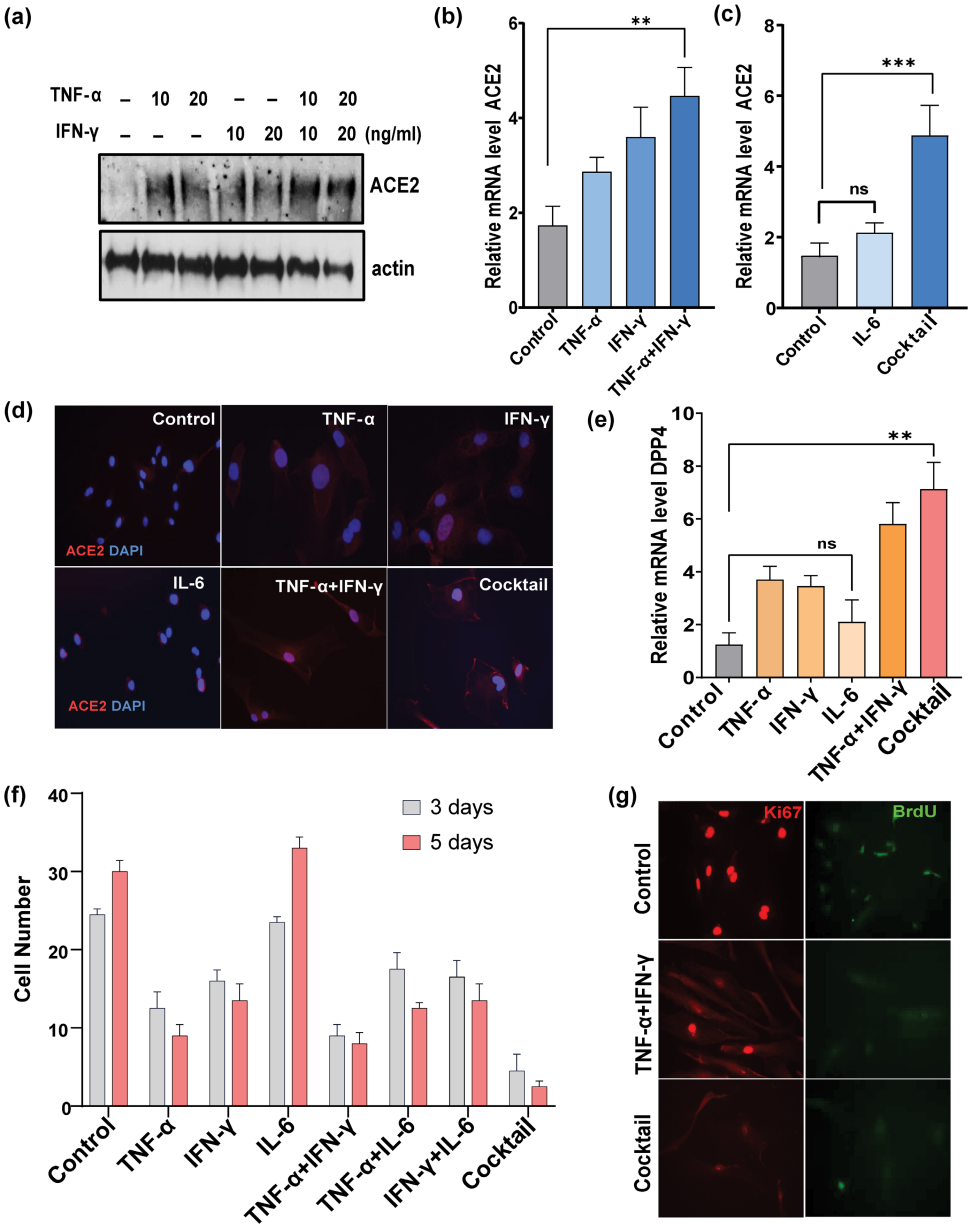
TNF‐α/IFN‐γ synergistically increase ACE2 and DPP4 and inhibit proliferation. (a) Western blot for ACE2 in HUVECs exposed to TNF‐α, IFN‐γ, or both for 3 days at the indicated concentrations. (b, c) relative expression of ACE2 in cells stimulated with TNF‐α, IFN‐β, IL‐6 or TNFα+IFN‐γ individually or as a cocktail (TNF‐α + IFN‐γ + IL‐6). (d) Immunostaining for cell surface expressed‐ACE2 (red) and DAPI (blue) in cells exposed to the indicated cytokines. DAPI staining indicates nuclear DNA. (e) Real‐time expression of DPP4 in cells exposed to the indicated cytokines. (f) Graph represents cell number as % of control after 3 and 5 days of stimulation with cytokines as indicated. (g) Immunostaining for the proliferation indicator, Ki67 (red), and the cell cycle marker, BrdU (green), in cells exposed to cytokines for 5 days. Data are representative of two (a, d, g) or three (b, c, e, f) independent experiments. Error bars show mean ± SD, *p < 0.05, **p < 0.01, ***p < 0.001; ns: Non‐significant

The expression of viral entry receptors and inflammation are important determinants of COVID‐19 infection and pathogenesis (Conde et al., 2020; Moore & June, 2020). Therefore, we tested effects of key inflammatory cytokines linked to COVID‐19 infection on SARS‐CoV2 receptors in senescent and non‐senescent cells. HUVECs were exposed to TNF‐α or IFN‐ γ alone or in combination for 3 days. Western analysis revealed higher ACE2 protein in cells exposed to TNF‐α combined with IFN‐γ, while only a modest increase in expression of ACE2 was detected in cells exposed to either TNF‐α or IFN‐γ alone (Figure 2a). The combination of TNF‐α and IFN‐γ or the three cytokines, TNF‐α + IFN‐γ + IL‐6 (“cocktail”), upregulated ACE2 and DPP4 gene expression (Figure 2b, c). In contrast, cells exposed to IL‐6 alone did not have any major change in expression of these receptors (Figure 2c), indicating that IL‐6 requires combination with TNF‐α/IFN‐γ to augment ACE2 expression. Cell surface expression of ACE2 was confirmed by immunostaining after stimulation with the various cytokines (Figure 2d). It is known that pro‐inflammatory cytokines such as TNF‐α and IFN‐γ can cause proliferative arrest and cellular senescence in various cell types, including endothelial cells (Hubackova et al., 2016; Kandhaya‐Pillai et al., 2017). Here, we explored the combined effects of these cytokines on proliferative arrest. Compared to TNF‐α or IFN‐γ, the combination of both or exposure to the cocktail lowered proliferative capacity of HUVECs as evidenced by decreased cell number, development of flattened and enlarged morphology, reduced BrdU incorporation, and decreased expression of the proliferation marker, Ki67 (Figure S2, 2e–g). In contrast, IL‐6 exposure alone modestly increased proliferation compared to control. Notably, IL‐6 in combination with either TNF‐α or IFN‐γ decreased proliferation, suggesting that IL‐6 requires presence of TNF‐α or IFN‐γ to exert anti‐proliferative effects. These results provide evidence that TNF‐α and IFN‐γ synergistically enhances ACE2/DPP4 expression and contributes to proliferative arrest, while IL‐6 requires presence of TNF‐α/IFN‐γ for these effects.

### 2.3 High STAT1 amplifies inflammation

**FIGURE 3:**
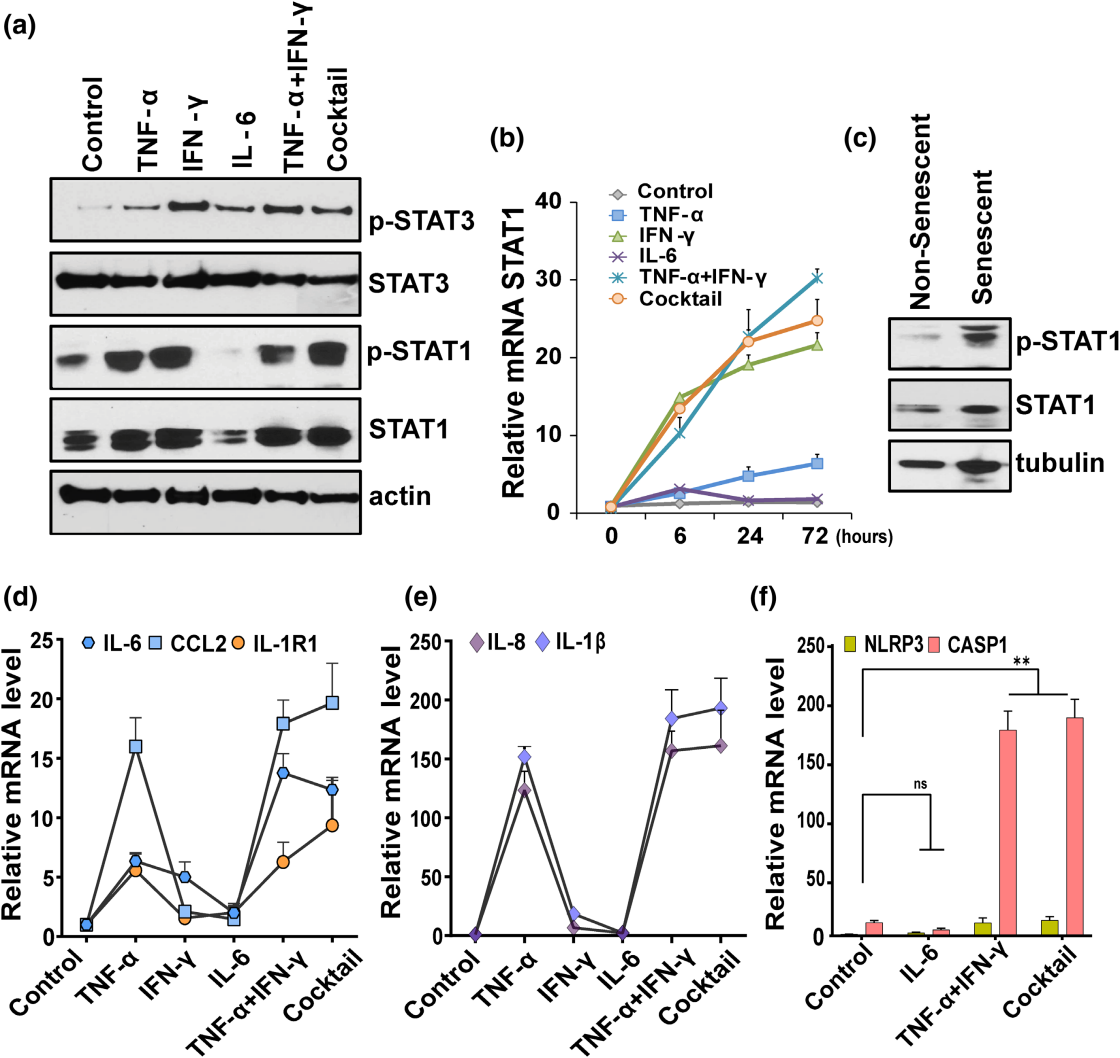
TNF‐α/IFN‐γ synergy accentuates inflammation in a STAT‐dependent manner. (a) Western blot analysis of pSTAT1, STAT1, pSTAT3, STAT3, and actin in HUVECs stimulated with the indicated cytokines for 3 days. (b) Time course analysis of STAT1 mRNA in cells treated with cytokines for the indicated times. (c) Western blot analysis of pSTAT1 and non‐phosphorylated STAT1 in non‐senescent and senescent HUVECs. Tubulin served as the control. (d–f) HUVECs were exposed to different cytokines for 3 days as indicated and qPCR performed for the SASP components, IL‐6, CCL2, IL‐1R1, IL‐8, and IL‐1β, and NLPR3 and CASP1. Data are representative of two (a–c) or three (b–f) independent experiments. Error bars show mean ± SD. *p < 0.05, **p < 0.01, and ***p < 0.001 by Student's *T*‐test

Multiple cytokines signal via the JAK/STAT pathway and its activation are linked to both cellular senescence and COVID‐19 infection (Kandhaya‐Pillai et al., 2017; Matsuyama et al., 2020; Xu et al., 2015; Yan et al., 2021). We therefore tested whether the JAK/STAT pathway plays a physiological role in cytokine‐induced ACE2/DPP4 receptor expression. We measured short‐ and long‐term phosphorylation status of STAT1/STAT3 in cells stimulated with cytokines. STAT3 phosphorylation was increased under all the conditions (Figure 3a, S3). Phosphorylation of STAT3 in response to IFN‐γ was higher than other conditions, supporting its role as an upstream regulator. Overall, the phosphorylation dynamics of STAT1 were distinct from those of STAT3. Time‐course experiments showed that TNF‐α or IFN‐γ alone, the combination of TNF‐α and IFN‐γ, or the cocktail tended to increase STAT1 phosphorylation. This peaked after 6 h and persisted even after 72 h of stimulation (Figure 3a, b). On the other hand, treatment with IL‐6 alone mediated transient phosphorylation of STAT1 that peaked earlier and returned to baseline after 72 h of exposure. We observed significant increases in STAT1 phosphorylation in late passage or replicatively senescent HUVECs compared to non‐senescent cells (Figure 3c), indicating a role for STAT1 in cell cycle arrest. Severe COVID‐19 is associated with uncontrolled and exaggerated inflammation (Moore & June, 2020; Zhang et al., 2020). In our model, the combination of TNF‐α and IFN‐γ or the cocktail induced amplified expression of the SASP components IL‐6, CCL2, IL‐8, IL‐R1, and IL‐1, mirroring the hyper‐inflammatory state of COVID‐19 (Figure 3d, e). Conversely, cells treated with IL‐6 alone failed to exhibit further induction of these cytokines. Cell exposed to TNF‐α + IFN‐γ or the cocktail significantly upregulated key inflammasomal genes, including NLRP3 and caspase‐1 (Figure 3f), consistent with a role for inflammasomal complexes in the hyper‐inflammatory response. No significant changes in inflammasome gene expression were seen in IL‐6 treated cells. Together, our experiments are consistent with the notion that synergy of TNF‐α/IFN‐γ‐induced excessive STAT1 activation has a role in amplifying, prolonging, and sustaining inflammation.

### 2.4 Blocking the JAK/STAT pathway reduces ACE2/DPP4 expression and controls inflammation

**FIGURE 4:**
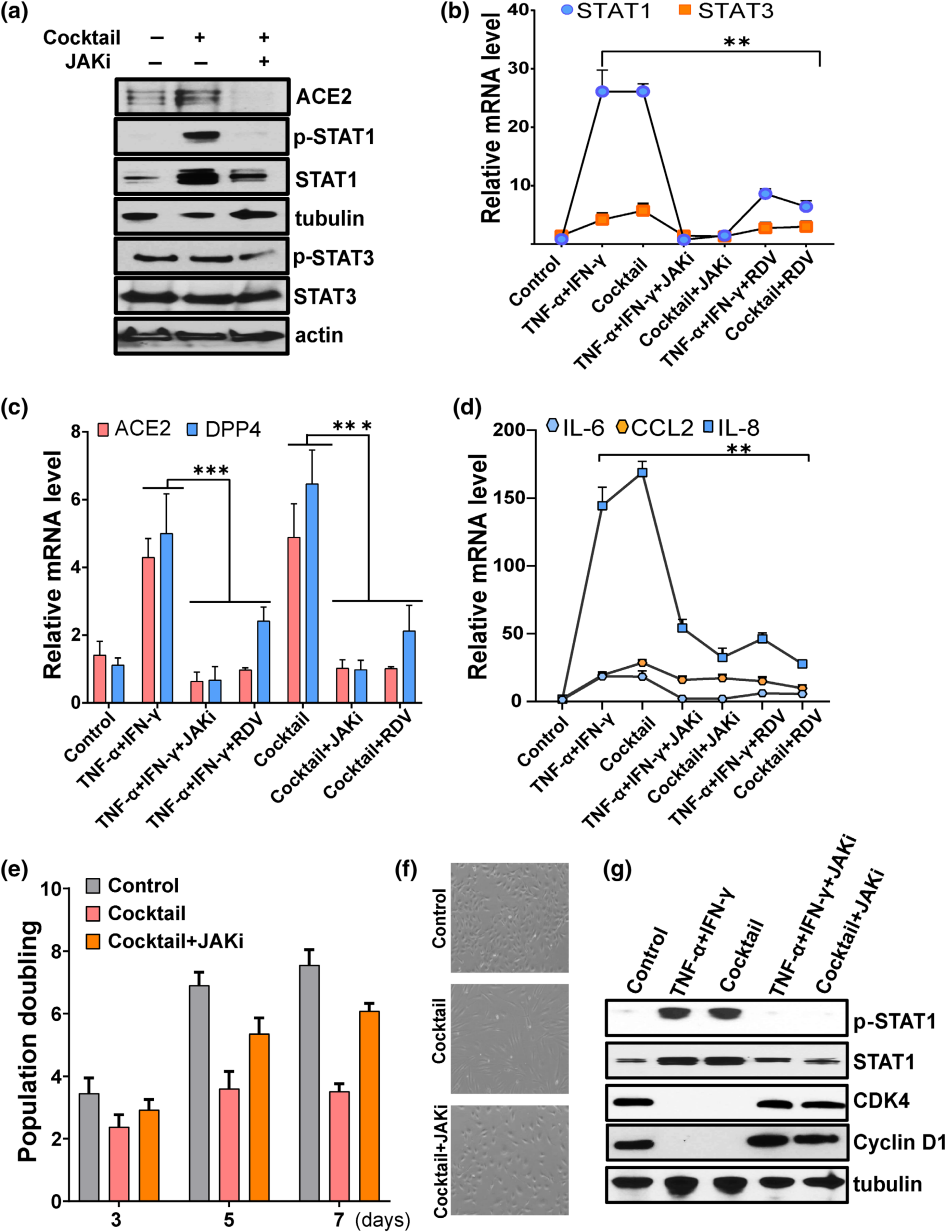
Targeting the JAK/STAT pathway reduces expression of SARS‐CoV‐2 viral entry receptors and suppresses inflammation. HUVECs were exposed to the indicated cytokines or vehicle or pretreated with ruxolitinib (1 μM) (JAKi) or remdesivir (2 μM) (RDV) for 30 min prior to cytokine stimulation. (a) Western blot analysis of ACE2, pSTAT1, STAT1, pSTAT3, STAT3, actin, and tubulin in control or cocktail or cocktail with JAK inhibitor. (b–d) Analysis of STAT1, STAT3, ACE2, DPP4, IL‐6, CCL2, and IL‐8 mRNA in control or cells treated with cytokines as indicated. (e) Cellular proliferation of control cells or cells exposed cocktail alone or with JAK inhibitor (1 μM) for the number of days indicated. (f) Representative phase contrast microscopic images of control or cells treated with cocktail or with JAK inhibitor. (g) Western blot analysis of pSTAT1, STAT1, CDK4, and cyclin D1 in cells exposed to the combination of TNF‐α + IFN‐γ or the cocktail or with the JAK inhibitor, ruxolitinib. Data are representative of two (a), two (g), or three (b‐f) independent experiments. Error bars show mean ± SD. *p < 0.05, **p < 0.01, or ***p < 0.001

Our results suggest a role for JAK/STAT1 in the regulation of SARS‐CoV‐2 receptor. To test this further, we targeted the JAK/STAT pathway using the FDA‐approved JAK inhibitor (JAKi), ruxolitinib, and analyzed effects of ruxolitinib on cytokine‐induced viral receptor activation. To do so, cells were cultured in the presence of cytokine cocktail or vehicle with or without ruxolitinib. Ruxolitinib normalized cytokine‐induced ACE2 and blunted phosphorylation of both STAT1 and STAT3 (Figure 4a), confirming a correlation between JAK/STAT and SARS‐CoV‐2 receptor activation. We observed treatment with JAKi, and the antiviral agent, remdesivir (RDV), decreased STAT1/3, which correlated with reduced expression of both ACE2 and DPP4 (Figure 4b, c). Expression of the key SASP factors, IL‐6, IL‐8, and CCL2, also decreased in the presence JAKi or RDV (Figure 4d). Treatment with JAKi prior to TNF‐α/IFN‐γ stimulation also prevented replicative arrest, as evidenced by retained proliferative capacity and cell cycle regulators, including cyclin D1 and CDK4 (Figure 4e–g). Overall, both ruxolitinib and RDV exhibited similar effects in suppressing the SASP and ACE2/DPP4 expression, although compared to JAKi, RDV was less effective at suppressing STAT1. This was reflected in a less significant effect on proliferative capacity (data not shown). These data indicate that JAKi and RDV are inhibitors of SARS‐COV‐2 receptor expression and inflammation.

### 2.5 Synergy of TNF‐α/IFN‐γ initiates an inflammatory feedback loop via STAT

**FIGURE 5:**
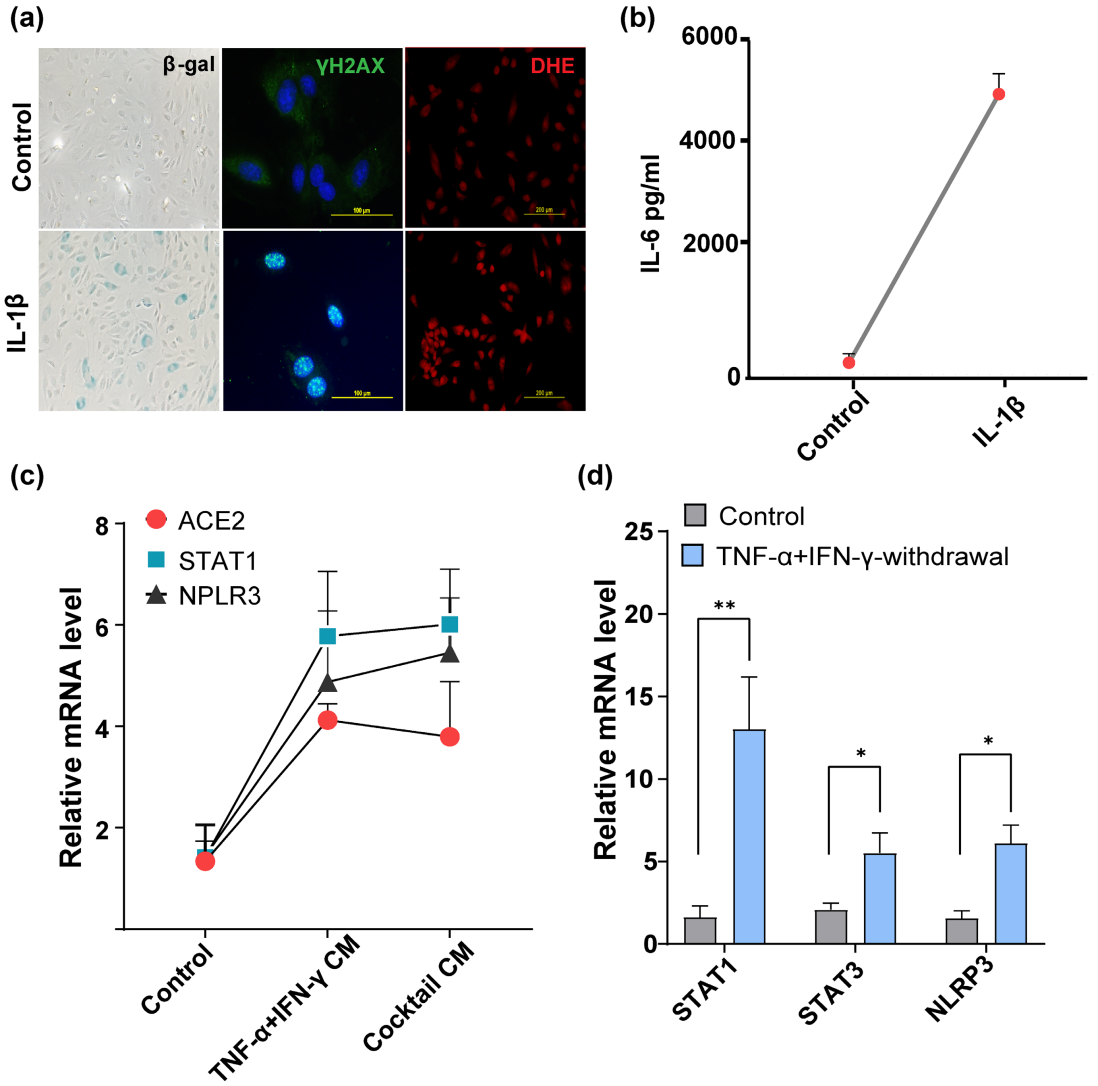
A positive feedback loop connects STAT and inflammation. (a) SA‐β‐gal, γ‐H2AX, and DHE (for ROS detection) staining in HUVECs stimulated with or without IL‐1β (20 ng/ml) for 48 h. (b) Quantification of IL‐6 production by ELISA in supernatants collected from cells cultured in the presence or absence of IL‐1β for 48 h. (c) Culture media from control cells or cells exposed to cytokines for 3 days were collected, centrifuged, filtered, and mixed with culture medium at a 1:2 ratio (culture supernatants: Culture medium). Cells were then treated with control conditioned medium or conditioned medium derived from cells exposed to cytokines for 24 h and analyzed for ACE2, STAT1, and NLRP3 mRNA. (d) Quantification of STAT1, STAT3, and NLRP3 mRNA in controls or 24 h after withdrawal of the cytokines, TNF‐α + IFN‐γ. data are representative of three (a–d) independent experiments. Error bars show mean ± SD. *p < 0.05, **p < 0.01, or ***p < 0.001

The pro‐inflammatory SASP is known to affect surrounding cells and reinforce senescence via autocrine or paracrine signals (Acosta et al., 2008). In particular, activation of the inflammasomal complex and secretion of IL‐1β could be downstream contributors to propagation of cellular senescence. Therefore, we tested effects IL‐1β on HUVECs. As shown in Figure 5a, b, exposure to IL‐1β accelerated endothelial replicative arrest, increased IL‐6 production, and increased DNA damage foci and ROS production. These data suggest secretion of IL‐1β by senescent cells, coupled with DNA damage, could be downstream stimulators of secondary senescence. To test further the paracrine effects of the cytokine‐induced SASP on ACE2 expression, we exposed proliferating HUVECs to conditioned medium collected from control or TNF‐α + IFN‐γ or cocktail‐treated cells. Cells exposed to conditioned medium derived from cytokine‐induced senescent cells exhibited upregulated ACE2 expression. This correlated with higher expression inflammatory genes, including STAT1 and NLRP3 (Figure 5c). Earlier, we reported that prolonged activation of STAT1/3 initiates a positive feedback loop that sustains the inflammatory network (Kandhaya‐Pillai et al., 2017). To determine whether a feed‐forward loop can be established with TNF‐α + IFN‐γ exposure, we tested STAT activity after withdrawal of cytokine stimuli. Expression of STAT1, STAT3, and the inflammasomal gene, NLRP3, persisted and was sustained even after removal of primary stress stimuli (Figure 5d). These data support our hypothesis that sustained activation of STAT and SASP establishes a feedback loop that could propagate replicative arrest and amplify inflammation.

## 3 DISCUSSION

Susceptibility to and adverse consequences from COVID‐19 increase exponentially with age (Bajaj et al., 2021; Davies et al., 2020; Zimmermann & Curtis, 2020). COVID‐related cytokine storm, characterized by an excessive inflammatory response, is a major threat to the elderly (Bartleson et al., 2021; Tisoncik et al., 2012; Zhang et al., 2020). Cell surface expression of SARS‐CoV‐2 viral entry receptors in host cells is important for viral infectivity and pathogenesis (Shang et al., 2020). We first addressed a basic question: are SARS‐CoV‐2 receptors expressed in senescent cells? We found that senescent cells do indeed express higher levels of ACE2, DPP4, and angiotensin II receptors than non‐senescent cultured HUVECs. Recent studies showed that higher nasal ACE2 expression in adults compared to children contributes to adverse outcomes in older adults (Baker et al., 2021; Bunyavanich et al., 2020; Conde et al., 2020). Despite the fact that SARS‐CoV‐2 uses ACE2 for entry, evidence supports the view that coronaviruses can engage other receptors to enter host cells (Trzaskalski et al., 2020; Valencia et al., 2020). It is likely that impaired immune responses plus the high abundance of ACE2/DPP4 in replicatively arrested senescent cells may contribute to higher probability of viral invasion in elderly or chronically ill compared to healthy younger individuals.

Our study links cytokine signaling to activation of SARS‐CoV‐2 receptors, inflammation, replicative arrest/ cellular senescence, and COVID‐19. Here, we describe the effects of TNF‐α, IFN‐γ, and IL‐6, all of which are linked to both cellular senescence and inflammation mediated by infections such as COVID‐19. Our experiments indicate a synergy between TNF‐α and IFN‐γ in inducing: (1) proliferative arrest, (2) high ACE2/DPP4 expression, (3) sustained activation of STAT1, and (4) augmented SASP‐related inflammation. These results suggest that increased cytokine levels contribute to increased expression of viral entry receptors that may contribute to progression to a hyper‐inflammatory state and complications of SARS‐CoV‐2 infection. The SASP signature after TNFα/IFN‐γ exposure, including IL‐6, IL‐8, CCL2, IL‐1β, and PAI‐1, overlaps the cytokine profile observed in severe cases of COVID‐19 (Bartleson et al., 2021; Moore & June, 2020). Karki et al. (2021) showed that synergism of TNF‐α and IFN‐γ triggers PANoptosis instead of senescence, which contradicts our findings. One possible explanation could be that cell fate decisions vary among cell types and inducing stimuli. In fact, in preadipocytes, we found differential effects of cytokines: TNF‐α alone increased preadipocyte proliferation, while TNF‐α/IFN‐γ synergy mediated DNA damage and the SASP with sustained JAK/STAT and mTOR expression (Figure S3). However, in agreement with other reports (Karki et al., 2021; Matsuyama et al., 2020), a combination of TNF‐α and IFN‐γ promoted prolonged STAT activation and augmented inflammation in both senescent preadipocytes and HUVECs.

Cellular senescence/ replicative arrest, inflammation, and COVID‐19 are all correlated with aging and chronic diseases (Bajaj et al., 2021; Bartleson et al., 2021; Bonafe et al., 2020; Camell et al., 2021; Domingues et al., 2020; Lee et al., 2021; Nehme & Borghesan, 2020; Salimi & Hamlyn, 2020). Heightened expression of ACE2 in senescent endothelial cells indicates there is a pathway to increased viral infection that contributes to endothelial injury, inflammation, and dysfunction in COVID‐19 (Bonaventura et al., 2021; Conde et al., 2020; Huertas et al., 2020; Jin et al., 2020; Varga et al., 2020). Viruses can induce cellular senescence. At the same time, viruses may exploit cellular senescence, since expression of viral receptors is increased in senescent cells (Kim et al., 2017; Kohli et al., 2021; Meyer et al., 2021; Seoane et al., 2020). Recently, viral infection, and in particular, SARS‐CoV‐2, has been shown to provoke cellular senescence and exacerbate the pro‐inflammatory SASP of senescent cells (Camell et al., 2021; Lee et al., 2021; Tripathi et al., 2021). It is conceivable that during SARS‐CoV‐2 infection, secretion of cytokines, specifically TNF‐α and IFN‐γ/‐IL‐6, mediates senescence and increases ACE2/DPP4 activity, which may in turn increase viral invasion, accentuate the SASP, and cause spread of senescence, leading to increased inflammation and cytokine storm. One recurrent observation was that IL‐6 alone fails to induce senescence, promote inflammation, or sustain STAT1 phosphorylation. However, it appears from our data that IL‐6 does contribute to downstream signaling since: (1) both TNF‐α and IFN‐γ significantly increase IL‐6 expression and (2) IL‐6 in conjunction with TNF‐α/IFN‐γ increases inflammation and decreases cells capable of proliferation. Thus, presence of IL‐6 may contribute to amplified and sustained inflammation. It will be interesting to examine the role of IL‐6 in detail in future studies.

**FIGURE 6:**
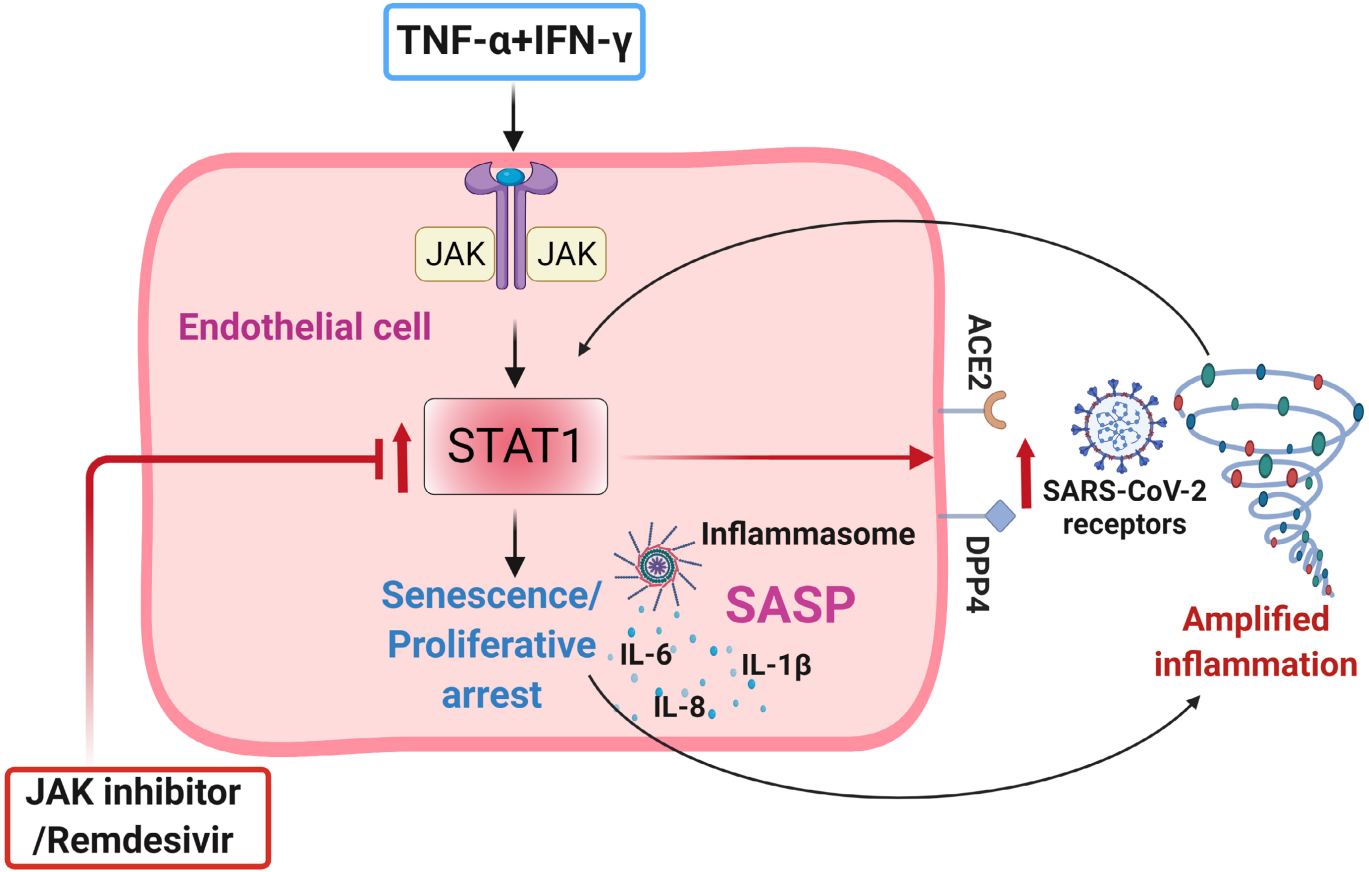
Schematic of TNF‐α + IFN‐γ‐mediated effect on endothelial cells. Exposure of HUVECs to TNF‐α + IFN‐γ increases SARS‐CoV‐2 receptor expression, accentuates the SASP, and induces senescence‐like proliferative arrest. Hyperactivated STAT1 coupled with the SASP and inflammasome creates a feedback loop, allowing sustained inflammation and spread of senescence. Targeting STAT1 with a JAK inhibitor (ruxolitinib), or the antiviral remdesivir, normalized ACE2/DPP4 expression and suppressed inflammation. Schematic sketch created with Biorender.com

The molecular connection between senescence and inflammation in viral infection is complex. JAK/STAT signaling is central to production of many cytokines and has been linked to inflammation, cellular senescence, and now COVID‐19 (Kandhaya‐Pillai et al., 2017; Matsuyama et al., 2020; Morris et al., 2018; Yan et al., 2020). Our study indicates that synergy between TNF‐α and IFN‐γ induces hyper‐activation of STAT1, inducing senescence‐associated hyper‐inflammation and activation of SARS‐CoV‐2 viral entry receptors (Figure 6). We found that, compared to either TNF‐α or IFN‐γ alone, the combination of these two cytokines increases STAT1, as reflected by higher expression of SASP factors. This extends and supports recent findings that hyper‐activated senescent cells promote spread of senescence and exacerbate inflammatory responses (Budamagunta et al., 2021). The use of JAK inhibitors in patients with COVID‐19 effectively reduces inflammation, increases survival, and prevents severe outcomes (Chen et al., 2021; Kalil et al., 2021; Rodriguez‐Garcia et al., 2020). We observed that treatment of cells with ruxolitinib blunted STAT1 and reduced cytokine‐mediated inflammation and ACE2/DPP4 expression. Inhibition of the STAT pathway not only controlled inflammation, but also normalized overall levels of cytokines and ACE2/DPP4. Despite our finding that RDV exerts slightly less effects on STAT than JAKi, both of these inhibitors reduced senescence‐associated inflammation and ACE2/DPP4 expression, supporting the contention that there is crosstalk with other mechanisms in regulating cytokine production.

As discussed above, inflammation, cellular senescence, and the pathogenesis of COVID‐19 are intimately connected, particularly in the context of aging and multiple underlying diseases. Based on our data, secreted factors from senescent cells that lead to increased expression of SARS‐CoV‐2 viral entry receptors may contribute to increasing senescent cell abundance and amplifying inflammation and cytokine storm. Inflammasome activation and increased IL‐1β could be downstream events that exacerbate cytokine secretion as well as cellular senescence, sustaining an inflammatory loop. Cytokine‐mediated senescence and associated inflammation promote and reinforce each another, leading to increased senescence and hyper‐inflammation, with a TNF‐α/IFNγ‐STAT1 axis being a link in this self‐amplifying senescence‐inflammation loop (Figure 6). Extrapolating our results and taking other recently published data into consideration, we propose a senescence‐associated cytokine loop could be a driver of cytokine storm in COVID‐19. Because STAT activation is crucial for regulation of both cellular senescence and inflammation, targeting STATs using JAK inhibitors may break this cycle. Drugs that broadly target senescent cells or intracellular cytokine signaling pathways could be effective strategies for controlling inflammatory circuits, especially in cases with severe inflammation. We and others have demonstrated the administration of senolytics to virally‐infected mice and cultured cells is a potential strategy for eliminating senescent cells and attenuating the SASP in β‐coronavirus infection (Camell et al., 2021; Lee et al., 2021). Clinical trials of both senolytics and JAKi for COVID‐19 are underway (Verdoorn et al., 2021). Our results suggest a molecular mechanism whereby cytokine‐driven cellular senescence is a link between inflammation and activation of SARS‐CoV‐2 viral entry receptors.

## 4 EXPERIMENTAL PROCEDURES

### 4.1 Cell culture

Human umbilical vein endothelial cells (HUVECs) were purchased from Lonza Bioscience (CC‐2519; Morristown, NJ, USA) and cultured in EGM‐2 medium supplemented with growth factors (EGM‐2 bullet kit; Clonetics/Lonza) following the manufacturer's instructions. HUVECs were maintained in 75cm^2^ or 25cm^2^ tissue culture flasks or 6 well plates kept at 37°C in a humidified incubator with 5% CO_2_ and 95% filtered air. For proliferation assays, 1 × 10^6^ HUVECs in 12 ml culture medium were seeded into 6‐well plates, 2 ml in each well, in triplicate. Cells were then treated with or without cytokines for the indicated times. Cell numbers were counted by hemocytometer in duplicate. Preadipocytes isolated from healthy donors were cultured as described previously (Zhu et al., 2015). All subjects gave informed consent. The protocol was approved by the Mayo Clinic Institutional Review Board for Human Research. Cells were maintained in Dulbecco's modified Eagle's medium, 10% fetal calf serum, and 1x penicillin/streptomycin in a 5% CO_2_, 37°C environment.

### 4.2 Cytokine stimulation and inhibitor assays

Recombinant human TNF‐α (315‐01A), IL‐6 (212–16), IFN‐γ (315–05), and IL‐1β (200‐01B) were purchased from Peprotech, Cranbury, NJ, and the JAK inhibitors, ruxolitinib (7064) and remdesivir (7226), from R&D Systems, Minneapolis, MN. Cells were stimulated with the cytokines IL‐6 (50 ng/ml), TNF‐α (20 ng/ml), and IFN‐γ (20 ng/ml) alone or a combination of TNF‐α + IFN‐γ (20 ng/ml of each) or all three (“cocktail” of IL‐6, TNF‐α, and IFN‐γ, 20 ng/ml of each) for 3, 5, or 7 days, unless indicated otherwise. For experiments with inhibitors, cells were cultured with or without JAK inhibitor (1 μM) or RDV (2 μM) for 30 min prior to cytokine stimulation. For conditioned medium (CM) experiments, cells were stimulated with or without cytokines for 3 days. Culture supernatants were then collected, centrifuged, filtered, and mixed with culture media at a 1:2 ratio. Early passage HUVECs were then cultured with control CM or cytokine‐induced senescent cell CM for 24 h.

### 4.3 Immunofluorescence staining

Immunofluorescence staining was performed as described previously (Renuka Kandhaya‐Pillai et al., 2021). Cells grown on sterilized cover slips were fixed with 3% formaldehyde in PBS at room temperature for 10 min, blocked with 10% fetal bovine serum in PBS for 30 min, and permeabilized with 0.2% Triton X‐100 and 3% BSA in PBS for 30 min. Cells were then incubated with primary antibodies against ACE2, Ki67, γ‐H2AX, or BrdU for 1 h and, after washing, stained with secondary antibody conjugated with Alexa‐fluor 488 or 583 for 30 min. Cells were washed three times with permeabilization buffer and once with PBS and then mounted in an anti‐fading mounting solution containing DAPI (P36966; Thermo Fisher Scientific, Waltham, MA, USA) on microscopic slides. For reactive oxygen species (ROS) detection, cells were grown on cover slips and treated with or without IL‐1β for 48 h, and then coverslips were collected and fixed with 4% paraformaldehyde. The fixed cells were labeled with 20 μM dihydroethidium (DHE; Molecular Probes, Carlsbad, CA, USA) for 30 min at 37°C and washed once with PBS prior to mounting onto slides. Images were acquired with a Leica wide‐field microscope with a 40× objective. All the images were captured at identical settings. Post‐acquisition analysis was performed using ImageJ.

### 4.4 RNA extraction, rtPCR, and Qpcr

Total cellular RNA was extracted from sub‐confluent cultures from the above‐mentioned experimental groups using tri‐reagent and stored at −80°C until processed. For RNA isolation, 200 μl chloroform were added to each sample, mixed vigorously, incubated at room temperature for 5 min, and centrifuged at 4°C at 12,000 g for 20 min. The upper aqueous phase was precipitated with isopropanol and RNA was pelleted with ethanol. The pellet was air dried for 15 min and 20 μl nuclease‐free water was added. RNA concentration and purity were analyzed using a Nanodrop spectrophotometer. cDNA was generated by reverse transcription carried out with 2 μg total RNA using a High‐Capacity cDNA Reverse Transcription Kit (4368814; Thermo Fisher) following the manufacturer's protocol. qPCR was performed using Taqman Fast Advanced master mix and Taqman probes in an Applied Biosystem 7500. Glyceraldehyde‐3‐phosphate dehydrogenase (GAPDH) was used as the internal control for all experiments. Three independent experiments were performed for every condition and technical duplicates or triplicates were studied for each reaction. Data were analyzed using the ΔΔ*C*_T_ method. Statistical significance was determined by Student's *T*‐test.

### 4.5 BrdU incorporation assay

The fraction of proliferating cells was determined by a 5‐bromo‐2deoxyuridine (BrdU) incorporation assay using a BrdU labeling and detection kit (11 296 736 001; Roche Applied Science, Indianapolis, IN, USA). To do so, cells were labeled for 48 h and processed according to the manufacturer's instructions.

### 4.6 Western blot analysis

For protein expression analysis, whole cell lysates were extracted using 2x Laemmeli sample buffer followed by sonication for 10 s. Equal amounts of protein extracts were resolved by SDS‐PAGE gel electrophoresis and transferred onto a nitrocellulose membrane and blocked with 5% non‐fat dry milk. The membranes were then immunoblotted with primary antibody overnight at 4°C followed by secondary antibody conjugated with horseradish peroxidase. The membrane was incubated with Signal Fire Elite ECL reagent according to the manufacturer's instructions. Signals were captured on AGFA (CP‐BU) films.

### 4.7 Senescence‐associated β‐galactosidase (SA‐β‐gal) staining

SA‐β‐gal activity was measured by using a Senescence β‐Galactosidase staining kit (9860; Cell Signaling Technology, Danvers, MA, USA) following the manufacturer's instructions. Briefly, the cells were washed twice with PBS, fixed with 1 ml of 1× fixing solution/ well (into each 35 mm^2^ well), and incubated at room temperature for 10 min. After removing fixation solution, the cells were washed twice with PBS and incubated overnight in 1 ml freshly prepared SA‐β‐gal staining solution/ well at 37°C overnight without CO_2_ and protected from light. The percent of cells positive for SA‐β‐gal was determined in 200 cells/ sample in triplicate.

### 4.8 Enzyme‐linked immunosorbent assay (ELISA)

Supernatants from cell cultures from control or cytokine‐treated cells were stored at −80°C until used. IL‐6 secreted into culture supernatants of control or treated cultures was quantified using a commercial ELISA kit according to the manufacturer's instructions (550,799 – OptEIA; BD Biosciences, San Diego, CA, USA).

### 4.9 Statistical analysis

Graphs were plotted using Prism 9.0 (Graph Pad). Three independent biological experiments were performed for all qPCR assays. All experiments including immunostaining, quantification, and determining cell number (population doubling, BrdU, Ki67, ACE2, and SA‐β‐gal staining) were conducted in technical duplicates or triplicates from two or three independent groups. Comparisons among groups were made by Student's *T*‐test. Data represent the mean ± SD (standard deviation). Statistical significance of all data is presented as asterisks (*p < 0.05; **p < 0.01; ***p < 0.001).

#### AUTHOR CONTRIBUTORS

R.K.P. and J.O. conceptualized the work and developed methodology. R.K.P. performed the experiments, analyzed data, and wrote the manuscript, X.Y. contributed to protein, RNA, and immunofluorescence experiments. J.O., G.M.M., T.T., and J.L.K. contributed to writing, preparing, and editing the manuscript. All the authors read, edited, and approved the final version of the manuscript.

#### ACKNOWLEDEMENTS

We thank Ms. Yi‐Chieh Wu and Lin Lee for editorial and technical assistance. This work was supported in part by NIH grants R01CA210916 (G.M.M./J.O.) and R37AG13925 (T.T./J.L.K.).

## CONFLICT OF INTEREST

The authors declare that they have no conflict of interest.

## Supporting information




Appendix S1

## Data Availability

All data are included in the article and Supplemental Information are available from the authors upon request.
