# Supplementary material for: TNF‐α/IFN‐γ synergy amplifies senescence‐associated inflammation and SARS‐CoV‐2 receptor expression via hyper‐activated JAK/STAT1
Source: Aging Cell. 2022 May 30;21(6):e13646. doi: 10.1111/acel.13646 (PMC9197409; doi:10.1111/acel.13646)
Supplement: Supplementary file 1 — Appendix S1 [file ACEL-21-0-s001.docx]

**Supplementary Fig. 1**

**Expression of IL-6 and IL-8 in senescent and non-senescent HUVECs.** Real-time PCR analyses for expression of IL-6 and IL-8 in senescent and non-senescent HUVECs. Data are representative of two independent experiments. Error bars show mean ± SD.

**Supplementary Fig. 2**


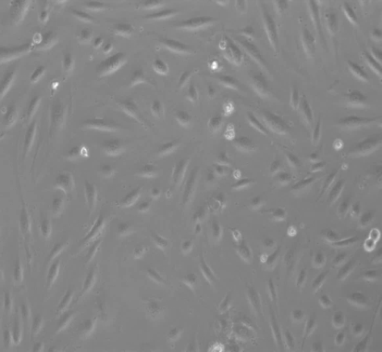

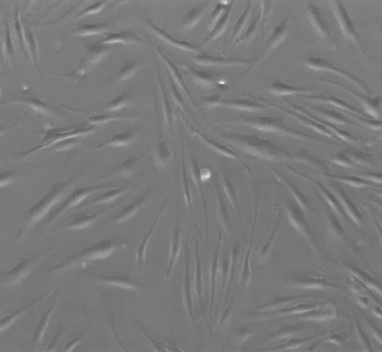

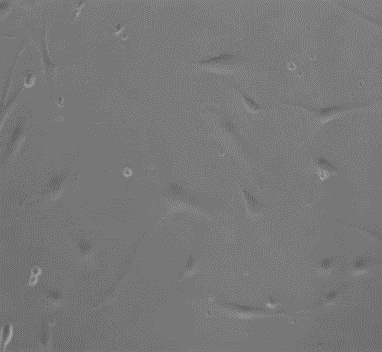

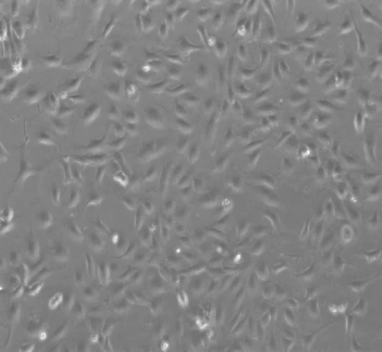

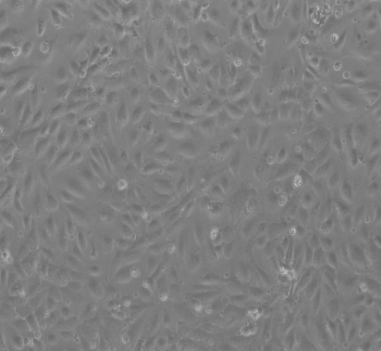

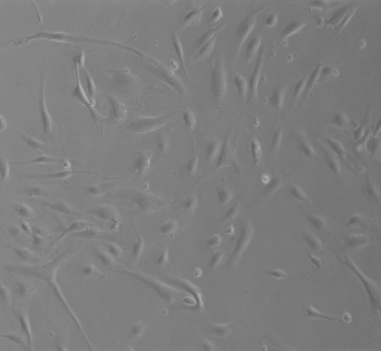


**Control**

**TNF-α**

**IL-6**

**IFN-γ**

**TNF-α+IFN-γ**

**Cocktail**

**Synergy of TNF-α/IFN-γ in mediating proliferative arrest.** Representative microscopic images of HUVECs treated with the indicated cytokines for 3 days. Cells exposed to TNF-α+IFN-γ or a cytokine cocktail (TNF-α+IFN-γ+IL-6) exhibited flattened and enlarged morphology.

**24 hrs**


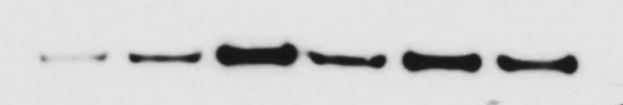


**p-STAT3**

**Control TNF- α**

**IFN-γ**

**IL-6**

**TNF-α+ IFN-γ**

**Cocktail**


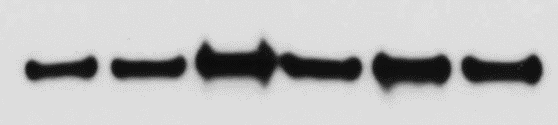


**STAT3**


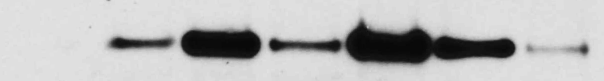

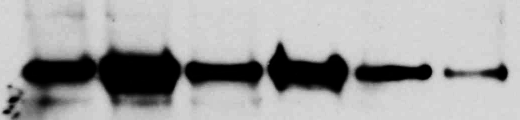


**p-STAT1**

**STAT1**

**Supplementary Fig. 3**

**Cytokine-mediated JAK/STAT activation.** Western blot analyses of pSTAT1, STAT1, pSTAT3, and STAT3 proteins in HUVECs stimulated with the indicated cytokines for 24 hours.

**a**

**b**

**Control**

**TNF-α**

**TNF-α+IFNγ**


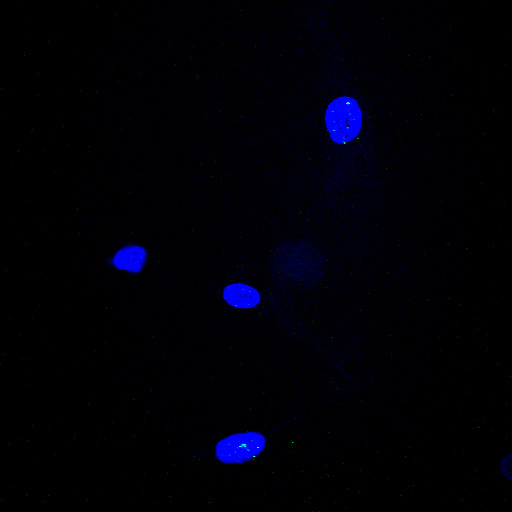

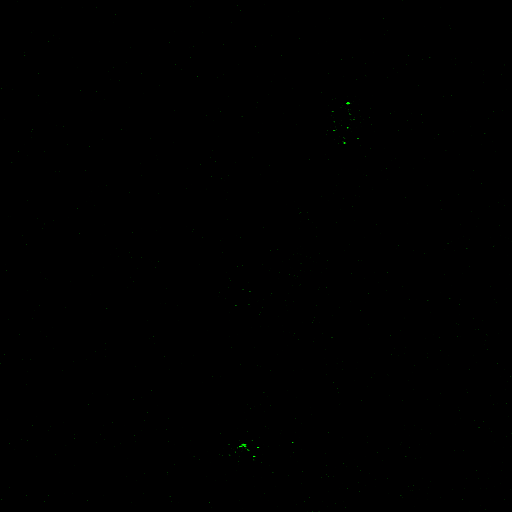


**Control**


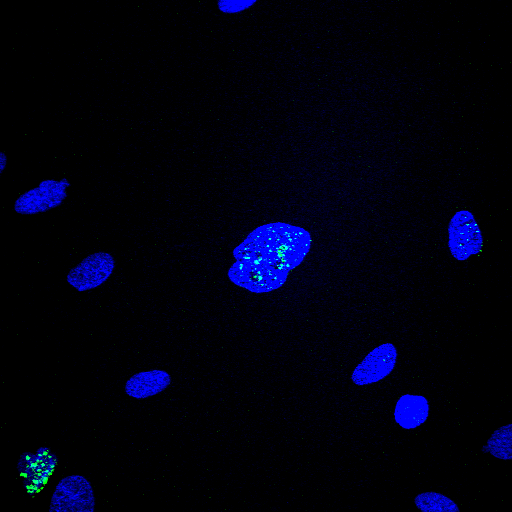

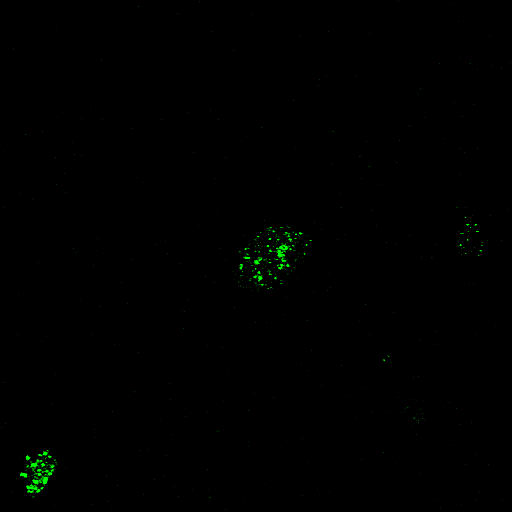


**TNF-α+IFN-γ**

**γ-H2AX**

**DAPI**

**c**


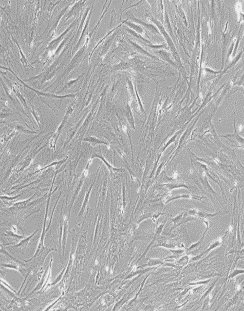

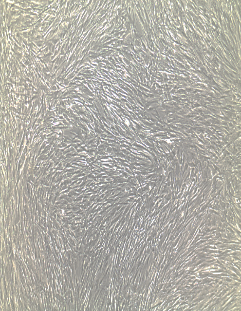

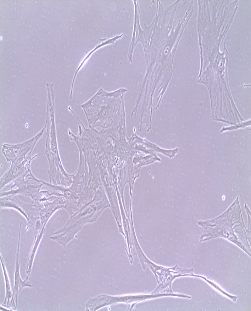


**Preadipoctyes**

**TNF-α - + - +**

**IFN-γ - - + +**

**p-STAT3**

**STAT3**

**p-STAT1**

**STAT1**


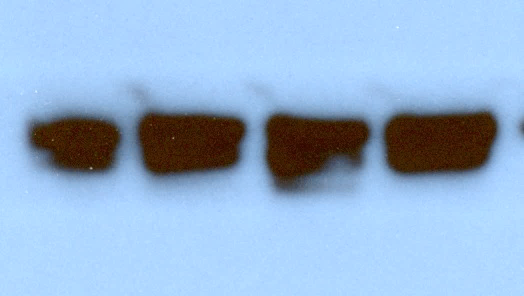

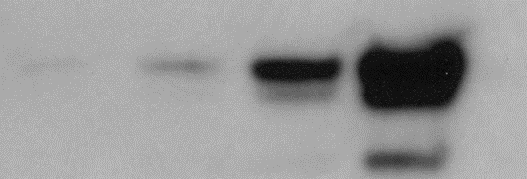

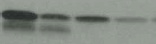

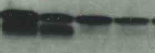

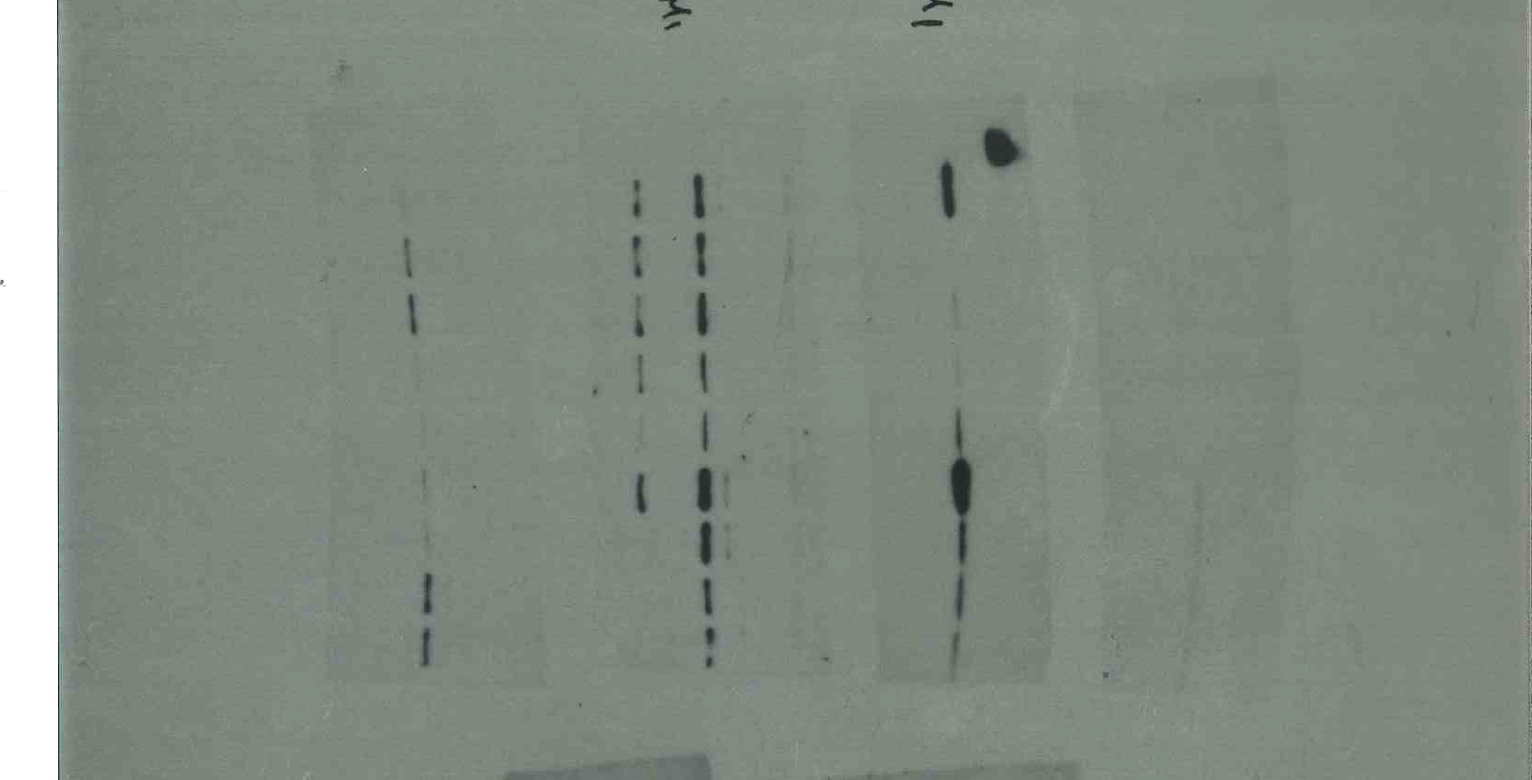

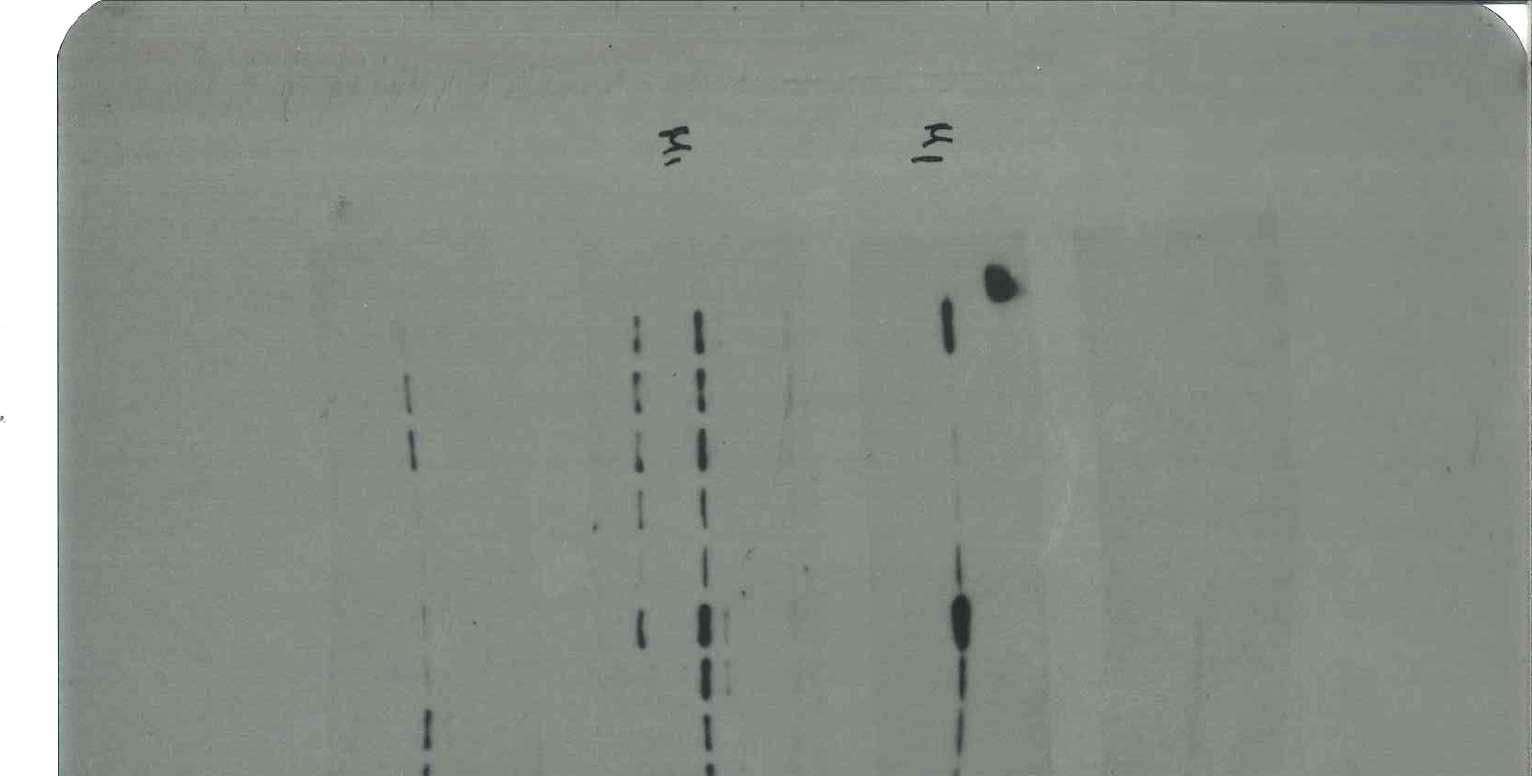


**p-mTOR**

**mTOR**

**Supplementary Fig. 4**

**DNA damage and prolonged activation of JAK/STAT and mTOR in TNF-α+IFN-γ-induced senescence.** a) Representative microscopic images of preadipocytes treated with or without TNF-α (20ng/ml) or TNFα+IFN-γ (50 ng/ml) for 15 days. Cells cultured with the combination of TNF-α+IFN-γ exhibited enlarged and flattened senescent cell morphology. b) Confocal images of immunostained cells treated with or without TNF-α+IFN-γ for 15 days and γ-H2A.X and nuclear staining with DAPI. The combination of TNF-α+IFN-γ increased γ-H2A.X foci compared to untreated or control cells. c) Western blot analysis of p-STAT1, STAT1, p-STAT3, STAT3, p-mTOR, and mTOR in preadipocytes cultured with TNF-α, IFN-γ, or the combination of both for 15 days.

| **Supplementary Table 1. Primers used for qPCR** | |  |
| --- | --- | --- |
| **Primers** | **Catalog # (Thermo Fisher)** | |
| Hs01085330_m1 -ACE2 | | 4351372 |
| Hs01122322_m1 -TMPRSS2 | | 4453320 |
| Hs01014005_m1 -STAT1 | | 4331182 |
| Hs06627156_sH -STAT3 | | 4448892 |
| Hs02786624_g1 -GAPDH | | 4351372 |
| Hs00991010_m1-IL1R1 | | 4453320 |
| Hs99999029_m1 -IL-1β | | 4453320 |
| Hs00897405_m1 -DPP4 | | 4351372 |
| Hs00354836_m1 -CASP1 | | 4331182 |
| Hs00167155_m1 -SERPINE1(PAI-1) | | 4453320 |
| Hs00918082_m1 -NLRP3 | | 4331182 |
| [Hs00171042_m1 -CXCL10](https://www.thermofisher.com/taqman-gene-expression/product/Hs00171042_m1?CID=&ICID=&subtype=) | | [4331182](https://www.thermofisher.com/order/catalog/product/4331182) |
| Hs03929033_u1 -IL-6 | | 4331182 |
| Hs00234140_m1 -CCL2 (MCP-1) | | 4331182 |
| Hs00174103_m1 -CXCL8 | | 4331182 |
| Hs01113624_g1 -TNF | | 4331182 |
| Hs00923894_m1 -P16 | | 4331182 |
| Hs00355782 m1 -p21CDKN1A | | 4331182 |
| Hs00986757_m1 -IFI16 | | 4331182 |
| Hs02621316_s1 -AGTR2 | | 4331182 |
| Hs05043708_s1 -AGTR1 | | 4448892 |
| Hs05456607_s1 -IRF1 | | 4351372 |
| Hs01032434_m1 -MKI67 | | 4331182 |

| **Supplementary Table 2. Antibodies used for Western blot** | |  |  |
| --- | --- | --- | --- |
| **Protein** | **Dilution** | **Catalog #** | **Vendor** |
| ACE2 | (1:1000) in milk TBS-T | AF933-SP | R&D Systems |
| ACE2 | (1:500) in milk TBS-T | 4355S | Cell Signaling |
| DPP4/CD26 (D6D8K) Rabbit mAb | (1:1000) in milk TBS-T | 67138T | Cell Signaling |
| [AGTR1 Ab](https://www.novusbio.com/products/agtr-1-antibody_nbp1-77078) | (1:1000) in milk TBS-T | [77078SS](https://www.novusbio.com/products/agtr-1-antibody_nbp1-77078) | Novus Biologicals |
| [p16 INK4A (D3W8G) Rabbit mAb](https://www.cellsignal.com/products/primary-antibodies/p16-ink4a-d3w8g-rabbit-mab/92803) | (1:1000) in milk TBS-T | 92803S | Cell Signaling |
| Phospho-Stat3 (Tyr705) (D3A7) Rabbit mAb | (1:1000) in milk TBS-T | 9145T | Cell Signaling |
| Stat3 (124H6) Mouse mAb | (1:1000) in milk TBS-T | 9139T | Cell Signaling |
| [Phospho-Stat1 (Ser727) Ab](https://www.cellsignal.com/products/primary-antibodies/phospho-stat1-ser727-antibody/9177) | (1:1000) in milk TBS-T | 9177S | Cell Signaling |
| [Stat1 Mouse mAb](https://www.cellsignal.com/products/primary-antibodies/stat1-antibody/9172) | (1:1000) in milk TBS-T | 9172T | Cell Signaling |
| CDK4 (D9G3E) Rabbit mAb | (1:1000) in milk TBS-T | 12790T | Cell Signaling |
| Cyclin D1 (E3P5S) Rabbit mAb | (1:1000) in milk TBS-T | 55506T | Cell Signaling |
| Anti-β-Actin | (1:5000) in milk TBS-T | A-5441 | Sigma |
| Anti- β-Tubulin | (1:2000) in milk TBS-T | T-4026 | Sigma |
| Secondary Anti-mouse IgG, HRP-linked Ab | (1:1000) in milk TBS-T | 7076S | Cell Signaling |
| Secondary Anti-rabbit IgG, HRP-linked Ab | (1:1000) in milk TBS-T | 7074S | Cell Signaling |
|  |  |  |  |
